# Plasma structuring within an expanded polar cap and cusp studied with the SS-520-3 sounding rocket

**DOI:** 10.1186/s40623-025-02189-7

**Published:** 2025-05-26

**Authors:** L. M. Buschmann, K. Asamura, L. B. N. Clausen, Y. Jin, H. Kojima, A. Kumamoto, S. Kurita, Y. Ogawa, K. Oksavik, Y. Saito, A. Spicher, S. Yokota, W. J. Miloch

**Affiliations:** 1 Department of Physics, University of Oslo, Postboks 1048, 0316 Oslo, Norway; 2 Institute of Space and Astronautical Science, Japan Aerospace Exploration Agency, 3-1-1 Yoshinodai, Chuo, Sagamihara, Kanagawa 252-5210 Japan; 3 Research Institute for Sustainable Humanosphere, Kyoto University, Uji, Japan; 4 Graduate School of Science, Tohoku University, Sendai, 980-8578 Japan; 5 National Institute of Polar Research (NIPR), Tachikawa, Tokyo 190-8518 Japan; 6 Department of Physics and Technology, University of Bergen, P.B. 7803, NO-5020 Bergen, Norway; 7 Department of Physics and Technology, UiT - The Arctic University of Norway, Klokkargårdsbakken 35, 9019 Tromsø, Norway

**Keywords:** Sounding rockets, Tongue of ionization, In-situ measurements, Cusp, Polar cap, Ionosphere

## Abstract

**Abstract:**

The SS-520-3 sounding rocket was launched on November 4th, 2021 as part of the Grand Challenge Initiative - Cusp from Ny-Ålesund, Svalbard. The rocket was launched into the cusp ionosphere during the main phase of a geomagnetic storm. In this study we utilize two low energy particle analyzers as well as a multi-needle Langmuir probe and an impedance probe as part of the rocket payload. This study aims to provide an overview of the flight conditions from a range of ground-based instruments and scintillation receivers. We were able to confirm that the rocket entered the cusp through the poleward edge at around $74^{\circ }$ of northern geographic latitude. Additionally, the rocket encountered polar cap patches (PCP), as well as a patch within the cusp (CP) and a newly-formed tongue of ionisation (TOI). Analysis of the density variations within different scale sizes show enhancements within meter-size and kilometer-size scales on the edges of PCP, within the CP and TOI. Overall, the enhancements within the variations on all sizes, as well as enhancements of the electron density were significantly higher within the CP and TOI in comparison to the PCP, though all structures were encountered at similar altitudes. The strongest enhancements were found on the poleward edge of the TOI, corresponding to strong fluctuations within the electron density. The TOI also had the largest enhancements within gradients of kilometer-size in comparison to meter-sizes. As the TOI is convecting with respect to the background plasma, the edges are susceptible to instabilities like the Kelvin–Helmholtz instability (KHI) and Gradient-Drift instability (GDI), giving rise to plasma density structures on several scale sizes.

**Graphical Abstract:**

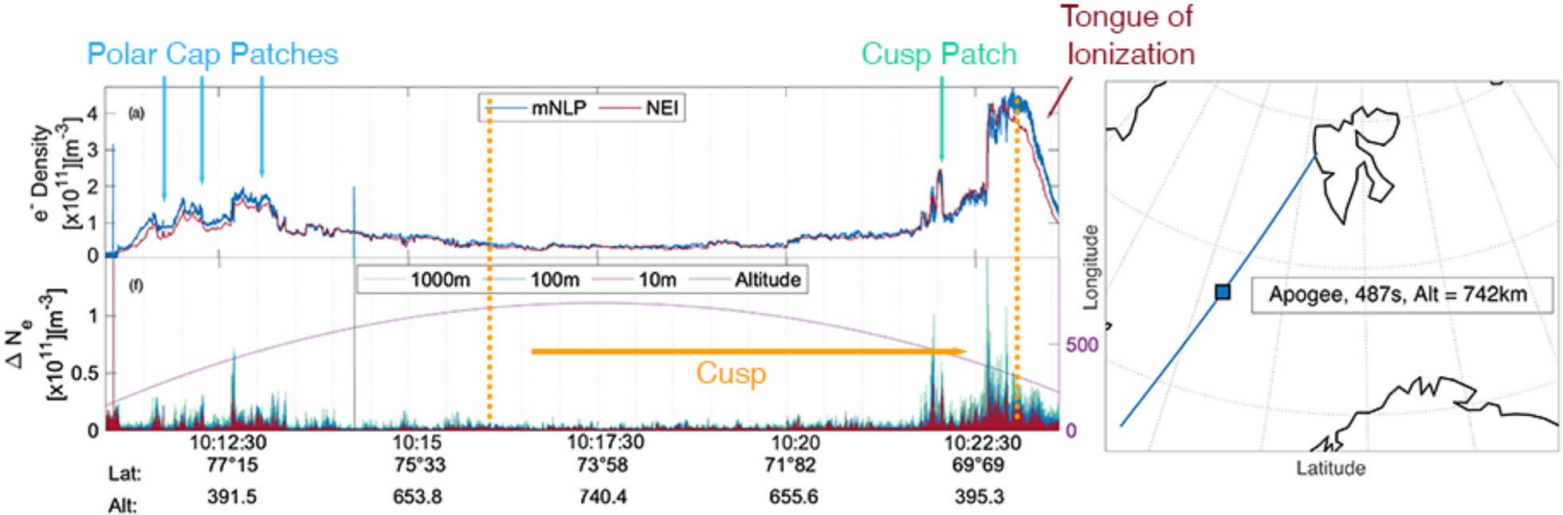

## Introduction

The solar wind consisting of charged particles that are emitted from the Sun’s corona can have a strong impact on the dynamics of the polar ionosphere. When the plasma of the solar wind reaches the Earth’s magnetic field, the field lines frozen in within the solar wind, also called interplanetary magnetic field (IMF), can reconnect with the terrestrial magnetic field lines. The newly reconnected field line then moves antisunward over the polar region, until it can reconnect on the nightside of the Earth in a process called nightside reconnection (Dungey 1961). For a southward IMF, the footprints of reconnected field lines can form a two-cell convection pattern in the high-latitude ionosphere, and the currents within aide convection of high-density dayside plasma over the polar cap into the nightside. The dusk-cell of this convection pattern is rotating clockwise, while the dawn-cell presents counter-clockwise convection (Heppner and Maynard 1987; Ruohoniemi and Greenwald 1996). However, this process is highly dynamic and can vary from the just described model. Particles with sufficient energy that follow along the reconnected field lines can then enter the ionosphere along the magnetic field and function as a source of free energy. This causes an onset of irregularities in the plasma density on various scales (Tsunoda 1988; Basu et al. 1990). The source of precipitating particles can stem from trapped particles, gyrating along the magnetic field lines until they gain sufficient energy to enter the ionosphere. Alternatively, particles can enter the ionosphere directly through a funnel-like shape called the cusp (Moen et al. 1996; Oksavik et al. 2000). Magnetosheath plasma has a direct access into the ionosphere through the cusp, and vice versa. Moreover, as the cusp location depends on the open-closed field line boundary (OCB), it serves as a footprint of dayside reconnection. Particle precipitation entering into the cusp ionosphere can cause large-scale irregularities with long life-times (Kelley et al. 1982; Moen et al. 2012, 2013; Buschmann et al. 2023).

Dayside plasma, characterized by high electron densities due to the impact of solar extreme ultraviolet (EUV) radiation, can travel poleward and procure a longitudinal narrow band that travels within the convection cell towards the nightside of the Earth, called a tongue of ionization (TOI). This TOI is often associated with the formation of irregularities in the plasma (Sato 1959; Sato and Rourke 1964; Knudsen 1974; Foster et al. 2005; van der Meeren et al. 2014; David et al. 2016).

Additionally, large-scale structures can convect from the dayside ionosphere over the polar region into the nightside, they can form large islands of enhanced density stemming mainly from the enhancement in density due to solar extreme ultraviolet (EUV) radiation (Tsunoda 1988; Carlson 2012), though less dense patches can also be formed by particle precipitation (Pinnock et al. 1993; Rodger et al. 1994; Goodwin et al. 2015; Oksavik et al. 2006). These islands of enhanced density are called polar cap patches (PCP) in the case where they have at least two times the plasma density of the background plasma and are located within the polar cap (Crowley et al. 1996; Tsunoda 1988). Similar enhancements within the auroral region are called auroral blobs on the nightside. Within the cusp they can be called cusp patches instead (Jin et al. 2017). Though research has been ongoing for several decades, there is no single definition of a blob (Vickrey et al. 1980; Kelley et al. 1982; Rino et al. 1983; Weber et al. 1985; Tsunoda 1988; Basu et al. 1990; Crowley et al. 2000). In recent years a distinction into two types of auroral blobs have been made: one that stems from high-density PCP traveling from the polar cap into the auroral zone and thus has its origin elsewhere, and one that stems from particle precipitation and the formation of the blob within the auroral zone (Jin et al. 2016). Similar to the TOI, both PCP and blobs can provide favorable conditions for plasma instabilities (Tsunoda 1988; Basu et al. 1990; Jin et al. 2016).

Generally, large-scale structures can convect into the polar cap where they can be broken down into smaller structures due to a variety of different ionospheric instabilities (Moen et al. 2012). Two of these instabilities are the Kelvin–Helmholtz instability (KHI) and the Gradient-Drift Instability (GDI) (Keskinen and Ossakow 1983; Kintner and Seyler 1985; Keskinen et al. 1988; Tsunoda 1988; Basu et al. 1990). The Kelvin–Helmholtz instability is driven mainly by high-velocity shears within the plasma. When two regions of plasma flow at different velocities with respect to each other, the instabilities at the edges may lead to eddies intertwining the boundary between these regions. The GDI is instead dominant on the edges of polar cap patches due to the variation in plasma density between the moving patch and the background plasma. Recently, a two-step process has been suggested in which the KHI is first structuring plasma which enters the polar cap from the cusp alongside the convection cell. This structured plasma can then be further broken down by the GDI leading to small-scale structures within the ionospheric plasma (Carlson et al. 2007; Moen et al. 2013).

Generally, small-scale structures are known to impair the signal of trans-ionospheric Global Navigation Satellite Systems (GNSS). This impairment includes rapid fluctuations in the amplitude and phase of the signal, also known as scintillations (Carlson 2012; Yeh and Liu 1982; Kintner et al. 2007). Scintillations have been known to cause navigation errors and failure of signal acquisition (Yeh and Liu 1982; Kintner et al. 2007). Recently, it has also been shown that particle precipitation can lead to strong scintillations within the cusp aurora (Kinrade et al. 2012, 2013; Jin et al. 2015; Oksavik et al. 2015). In the last decades, human activity has risen in polar regions which depends on accurate location services for marine traffic and aviation services. Thus, there has been an increase in research on the formation plasma of irregularities in the polar ionosphere and its connection to scintillations within recent years (Kintner et al. 2007; Oksavik et al. 2012; Moen et al. 2013; Jin et al. 2014).

To study the plasma structuring and processes in the ionosphere in detail, sounding rockets, which allow for in situ measurements of plasma parameters, are often used. One of such rockets is the SS-520-3 sounding rocket. The rocket was launched during the main phase of a geomagnetic storm into the polar cap, before encountering an active cusp aurora. This paper aims to describe the background conditions of the launch from several ground-based and in situ data sets. The main instrument used in this study is the multi-needle Langmuir probe, an array of probes that can measure the electron density with a cadence of 3.2 kHz. Due to the high sampling rate we are able to analyze density fluctuations on several orders of magnitude, making it possible to compare variations in the density on several different spatial scales within PCP, cusp patches and the TOI.

This paper is organized as follows: a brief overview of the launch and a detailed description of the main instruments used are provided in Section 2. Section 3 focuses on the results, including the flight conditions and analyses of density, particle precipitation and slope analysis data. The results are then further discussed within Section 4, while a conclusion is provided in Section 5.

## Methodology

**Fig. 1:**
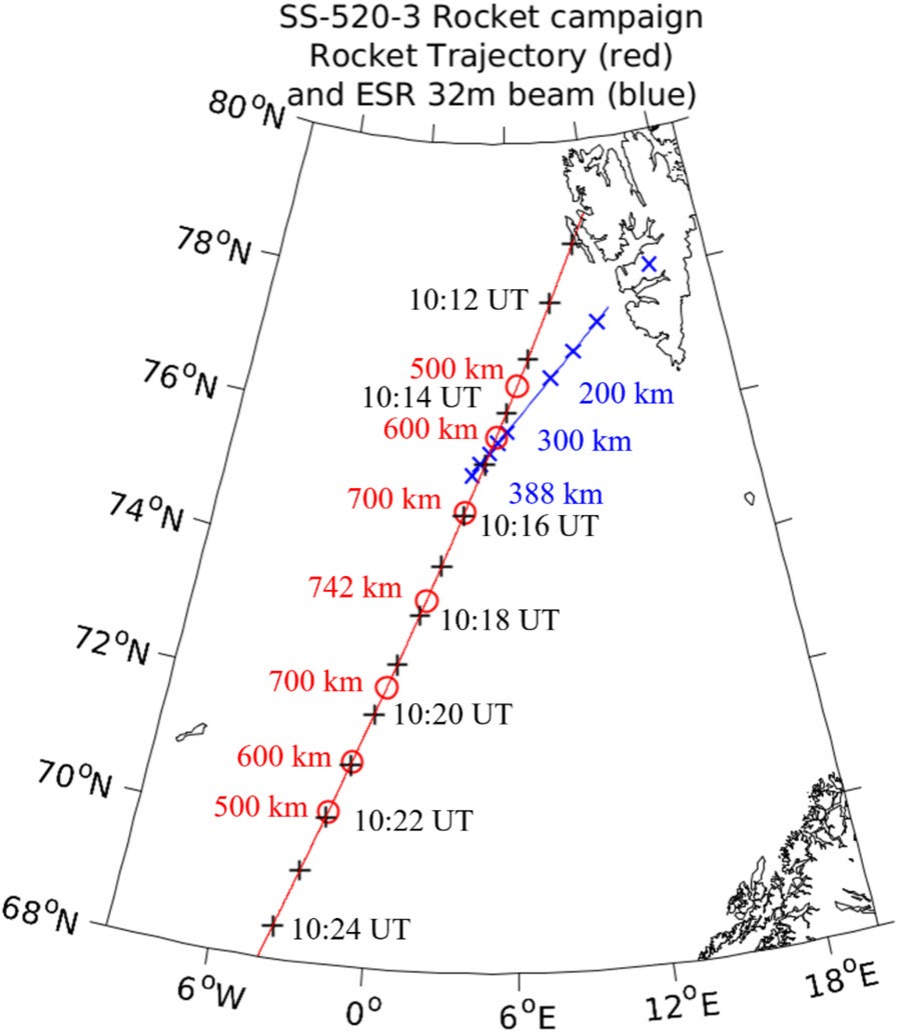
Trajectory of the SS-520-3 sounding rocket shown in red and field of view of the EISCAT 32 m beam indicated in blue with corresponding altitudes. The apex is reached 487 s after launch (corresponding to 10:17:31 UT), at an altitude of 742 km. The graph is presented in geographic coordinates

The SS-520-3 sounding rocket was launched on November 4th, 2021 at 10:09:25 UT from SvalRak located in Ny-Ålesund, Svalbard, Norway, at $78^{\circ }55'$ N, $11^{\circ }51'$ E as part of the Grand Challenge Initiative-Cusp (GCI-Cusp) (Moen et al. 2018). The rocket was launched into active cusp aurora with an apogee of 742 km that was reached 487 s into the flight corresponding to 10:17:32 UT. The rocket trajectory and the position of the apogee can be inferred from Figure 1. Note that all coordinates indicated with cardinal directions (N, E, S, W) within this manuscript are indicating geographic coordinates. All magnetic coordinates, if used, are indicated with magnetic latitude (MLat) and magnetic local time (MLT).

The main instruments utilized in this work contain the multi-needle Langmuir probe (mNLP) (Bekkeng et al. 2010; Jacobsen et al. 2010), the Number density measurement of Electron by Impedance (NEI) probe and the Low Energy Particle Analyzer (LEP) for ions and electrons, respectively.

The mNLP used in this mission consists of a set of four identical cylindrical Langmuir probes, each with a radius and length of 0.51 mm and 25 mm, respectively. The four probes were mounted on booms of 422 mm length. Two booms were protruding from the same structure on one side of the rocket with and angle of $27.54^{\circ }$ in between the probes. Another similar structure was mounted on the opposite side of the rocket. The booms in itself were angled at $9.94^{\circ }$ from the rocket axis towards the top of the rocket. This led to a distance of 236.26 mm between two probe tips on the same boom structure, and 317.54 mm between two tips on opposite sides of the rocket. This geometry located the probes in front of the rocket nose, thus minimizing wake effects on the probes. The probes were able to sample the electron density at a cadence of 3.2 kHz with applied biases of 3, 4.5, 6 and 7.5 V, respectively. The mNLP utilizes four cylindrical probes, the current drawn to each probe can be inferred from

1 $$\begin{aligned} I_c = n_eq\sqrt{\frac{kT_e}{2\pi m_e}}2\pi rl \frac{2}{\sqrt{\pi }}\left[ 1+\frac{q(V_f + V_b)}{kT_e}\right] ^{\beta }, \end{aligned}$$

where $T_e$, $n_e$, $m_e$ and *q* are the electron temperature, density, mass and charge, respectively, *k* is Boltzmann’s constant, *r* and *l* are the probe radius and length, respectively, and $V_f$ and $V_b$ are the floating or reference potential and the bias potential, respectively (Jacobsen et al. 2010; Bekkeng et al. 2010).

The factor $\beta$ depends on the probe geometry. For a finite-length probe with $l \gg \lambda _D$, $\beta$ equates 1/2. Although finite-length effects may lead to a variation of the factor, $\beta = 1/2$ has been found to be a good estimate ( Jacobsen et al. (2010); Hoang et al. (2018); Marholm and Marchand (2020); Guthrie et al. (2021)). For $\beta = 1/2$, the electron density $n_e$ can be rewritten from the above equation and for a system of probes inferred from the squared current to the probes over the voltage as:

2 $$\begin{aligned} n_e = \left( \frac{m_e}{2q(2qrl)^2} \frac{d(I_c^2)}{dV_b}\right) ^{\beta = \frac{1}{2}}. \end{aligned}$$

This equation was derived under the assumptions that the probe radius is much smaller in comparison to the Debye length, that the plasma follows a Maxwellian velocity distribution and is collisionless, non-drifting and unmagnetized (Jacobsen et al. 2010). In the F-region ionosphere at the rocket’s altitude, these criteria are generally fulfilled. The thermal velocity of the electrons is generally much larger than the drift velocity. Moreover, the electron mean free path is sufficiently long, the neutral density at these altitudes is comparably small, and the electron Larmor radius is much larger than the probe radius.

From Equation 2 one can see that if the squared collected currents are plotted against the probe biases, the electron density is proportional to a slope in this plot and the residual factor is only dependent on known factors. A different probe geometry will alter the value of $\beta$, which will thus affect the obtained electron density due to fluctuations in the electron temperature and floating potential (Marholm and Marchand 2020). The absolute density obtained with probes with a $\beta \ne 1/2$ has been found to be overestimated by 20–45% in some cases while simulations have found an overestimation by an order of magnitude (Hoang et al. 2018; Guthrie et al. 2021). As in this study we are mainly interested in fluctuations within the electron density, a constant overestimation of the electron density would not affect the results in any matter. In addition, we use an impedance probe to obtain another measure of the electron density.

Before obtaining the density from Equation 2, we removed the spin using a fast Fourier transform (FFT) and applied bandpass filters. The spin frequency was approximately 1 Hz, and thus we removed frequencies between 0.9 and 1.1 Hz and between 1.9 and 2.1 Hz, in order to remove the first and second harmonic frequencies. The calculated despun density was then detrended with a third order Savitzky–Golay filter for 10-s intervals in order to analyze the fluctuations within the electron density. The time interval for the filter was chosen in order to remove the background density, but it let km-sized fluctuations remain in the signal.

The NEI probe consists of a spherical probe with a diameter of 1.2 cm. In full deployment, the probe measures 1.2 m. The NEI utilizes a frequency sweep in order to find the upper hybrid resonance frequency of the electrons, from which the electron density can be calculated. A more detailed description of how to calculate the electron density from an impedance probe can be obtained from Wakabayashi et al. (2013). The probe has a sweep time of 125 ms, and thus a cadence of 8 Hz. A similar design of the NEI probe has been used on a variety of previous sounding rockets, and further specifications of the probe can be obtained from Suzuki et al. (2010), who have used the same front-end of NEI probe as used in this work.

The LEP instruments consist of two identical top-hat electrostatic analyzers (ESA) along two micro-channel plate (MCP) detectors, one for ions and one for electrons, respectively. The ESA used for this sounding rocket were equipped with an ultra-thin carbon foil between the ESA and the MCP detector, thus measuring the current generated by secondary electrons emitted from electron and ion impact, rather than electrons and ions directly. This ultra-thin carbon foil was added in order to develop an ESA that can measure both electrons and ions, simultaneously. For this mission, however, each ESA measured only one species. The specifications for both ESA and development on the system can be inferred from Yokota et al. (2024).

From the ground-based instruments we used the EISCAT Svalbard radar, located in Longyearbyen, Svalbard, consisting of two antennas. One is a fixed 42-m antenna oriented towards the local magnetic field line, measuring ionospheric parameters over Longyearbyen. The other, a steerable 32-m antenna, continued the fixed observations at a low elevation angle (36.0 deg), pointing south-west (azimuth angle of 229.7 deg), to measure changes in ionospheric parameters close to the rocket trajectory. Their real-time monitoring allows to measure the ionospheric conditions necessary to determine the launch of the SS-520-3 rocket. The radar observations were carried out as part of a peer-reviewed program and an international collaboration between Japan, Norway and Sweden. In this study, we utilize data from the 32-m antenna with a 60-s resolution.

We also use the total electron content (TEC) and scintillation data from The University of Bergen Global Navigation Satellite System Data Collection (Oksavik 2020a), and the University of Oslo receivers. Documentation of the data products is given by Oksavik (2020b).

Additionally, we use data of precipitating electrons from the Total Electron Detector (TED) and the Medium Energy Proton and Electron Detector (MEPED) from the NOAA-18 satellite (Evans 2000) and the MetOP3 satellite (Edwards et al. 2006). The NOAA-18 satellite is operated by the National Oceanic and Atmospheric Administration in a sun-synchronous orbit at around 852 km. The MetOP3-satellite operated by the European Space Agency (ESA) and the European Organisation for the Exploitation of Meteorological Satellites (EUMETSAT) is in a sun-synchronous orbit at around 817 km. Both satellites orbit the Earth roughly every 102 min. The TED measures particles at 2 different angles, along the local zenith ($0^{\circ }$) and $30^{\circ }$ from local zenith. We use four energy bands from the TED, which are presented in Table 1. The MEPED also measures particles at 2 angles, along the local zenith ($0^{\circ }$) and perpendicular to the local zenith ($90^{\circ }$). The MEPED collects energies in four energy bands centered around 40, 130, 287 and 612 keV. While both detectors measure electrons and protons, we only show the electron data in this study.

Furthermore, we use the TEC data obtained from the Madrigal database (Rideout and Coster 2006; Vierinen et al. 2016).

## Observations

### Background observations

In this section we first present observations depicting background conditions for the launch, which are followed by in situ measurements from the rocket.

**Fig. 2:**
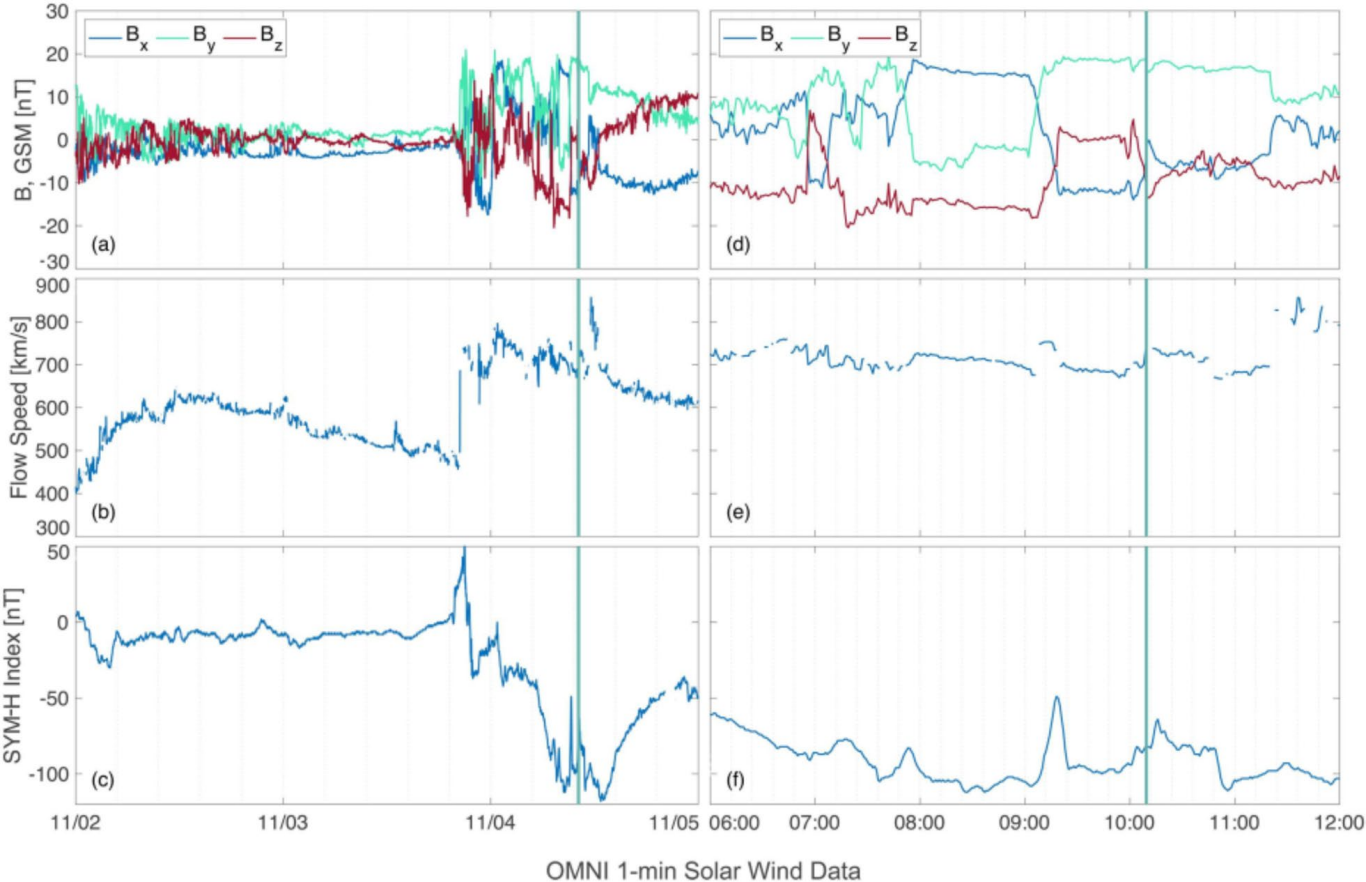
x, y and z-components of the interplanetary magnetic field (IMF) (**a**), solar wind flow speed (**b**) and Sym-H index (**c**) between November 2nd throughout November 5th. **d**, **e** and **f** show the same values in the same order but from 06:00 UT to 12:00 UT on November 4th, 2021. The magnetic field coordinates are given in GSM coordinates. The launch time is indicated with a vertical turquoise line

Figure 2 shows the interplanetary magnetic field (IMF) in geocentric solar magnetospheric (GSM) coordinates (panel a), the flow speed of the solar wind (panel b), and the Sym-H index (panel c) for the time between November 2nd throughout November 5th obtained from the 1-min NASA OMNI dataset through OMNIweb (Papitashvili et al. 2020). The launch time is indicated by a vertical turquoise line. Panels d, e and f show the same physical quantities for the time frame of 6:00 UT - 12:00 UT on November 4th. Note that the OMNI dataset is shifted to the bow shock.

During the time of launch, a geomagnetic storm occurred, as indicated in Fig. 2c. The Sym-H index drops late on November 3rd and reaches its lowest value of -118 nT just after noon on November 4th. The rocket launch thus corresponds to the main phase of the storm (Regi et al. 2022). According to Table 2, the storm can be classified as intense (with values below -100 nT). During the same time the solar wind flow speed (panels b and e) reaches values of about 700 m $\hbox {s}^{-1}$ at the time of the launch. Panel d shows the IMF around the launch time. Between 8:00 UT and 9:00 UT, $B_x$ reaches positive values of around 20 nT, while $B_y$ is weakly negative. $B_z$ is strongly negative with values of down to −20 nT. At 9:00 UT all three IMF coordinates change strongly. $B_x$ falls to negative values around −10 nT, while $B_z$ becomes weakly positive and $B_y$ reaches positive values up to 20 nT. While $B_y$ stays unchanged until after 11:00 UT, $B_x$ and $B_z$ change again just around launch. $B_x$ briefly reaches 0 nT before falling to weak negative values again for the duration of the launch. $B_z$ falls strongly to −15 nT and stays negative throughout the flight.

**Table 1:** Energy bands for the TED, given in electron volt (eV)

Low-energy Edge (eV)	Centre Value (eV)	High-energy Edge (eV)
154	189	224
688	844	1000
2115	2595	3075
6503	7980	9457

**Table 2:** Classification of storms according to the Sym-H index Gonzalez et al. (1994)

Geomagnetic storm	Sym-H index (nT)
Intense	−100 > Sym-H $\ge$ −250
Moderate	−50 > Sym-H $\ge$ −100
Weak	−30 > Sym-H $\ge$ −50

**Table 3:** Geographic coordinates of GNSS receiver stations

Station name	Geographic latitude and longitude
Bjørnøya (BJN)	$74.50^{\circ }\hbox {N}$, $19.00^{\circ }\hbox {E}$
Hopen (HOP)	$76.51^{\circ }\hbox {N}$, $25.01^{\circ }\hbox {E}$
Hornsund (HOR)	$77.00^{\circ }\hbox {N}$, $15.55^{\circ }\hbox {E}$
Longyearbyen (KHO)	$78.15^{\circ }\hbox {N}$, $16.04^{\circ }\hbox {E}$
Ny-Ålesund (NYA)	$78.92^{\circ }\hbox {N}$, $11.93^{\circ }\hbox {E}$

**Fig. 3:**
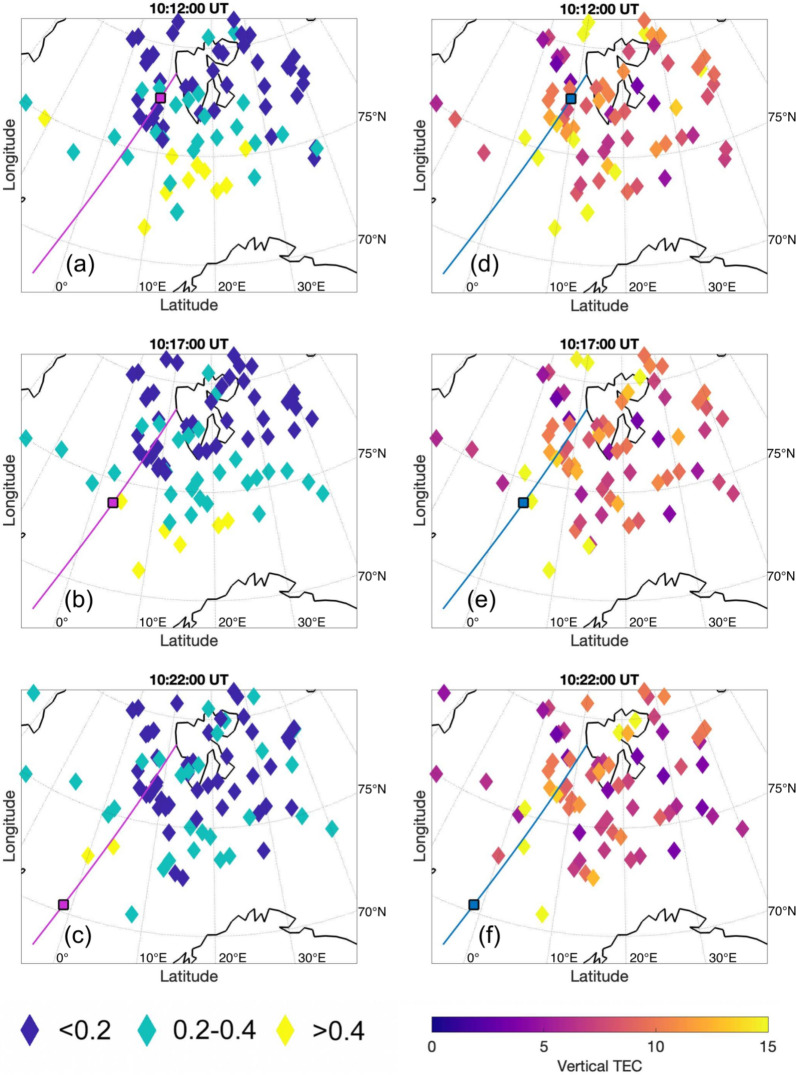
Scintillation indices $\sigma _\phi$ (**a**, **b** and **c**), and TEC data mapped to 250 km altitude (**d**, **e** and **f**) for 10:12 UT, 10:17 UT and 10:22 UT from top to bottom, respectively. The geographic coordinates of the GNSS receiver stations can be inferred from Table 3. The rhombus shapes indicate available data points. The rocket trajectory is indicated by a magenta line in the left column and by a blue line in the right column. The current rocket position is indicated by a magenta square in the left column and by a blue square in the right column

Figure 3 shows the $\sigma _\phi$ scintillation indices (panels a, b and c) and total electron content (TEC) data (panels d, e and f) mapped onto 250 km altitude for 10:12 UT, 10:17 UT and 10:22 UT from top to bottom, respectively. The rocket trajectory is indicated by a solid line (magenta in the left and blue in the right column). In each panel the rocket position is indicated by a magenta square in the left and by a blue square in the right column.

Panels a–c show the $\sigma \,_\phi$ indices. Generally, enhancements up to 0.4 units are located poleward of $75^{\circ }\hbox {N}$, with a tendency for larger indices further located towards the equator. Moreover, the largest enhancements can be found equatorward of $75^{\circ }\hbox {N}$ and between $0^{\circ }$ and $20^{\circ }\hbox {E}$. Overall, the highest phase scintillation indices seem to move towards the south-west when comparing panel a (10:12 UT) to panel b and c (10:17,UT and 10:22 UT, respectively). Note, that the graphs are presented in geographic coordinates.

Panels d–f show the TEC data in Vertical TEC (VTEC) units. While the majority of the signals have values between 0 and 15 VTEC units, some enhancements can be seen equatorward of $75^{\circ }\hbox {N}$ and between $0^{\circ }$ and $20^{\circ }\hbox {E}$. Additionally, strong enhancements can be seen above and poleward of Svalbard in varying locations throughout the flight.

**Fig. 4:**
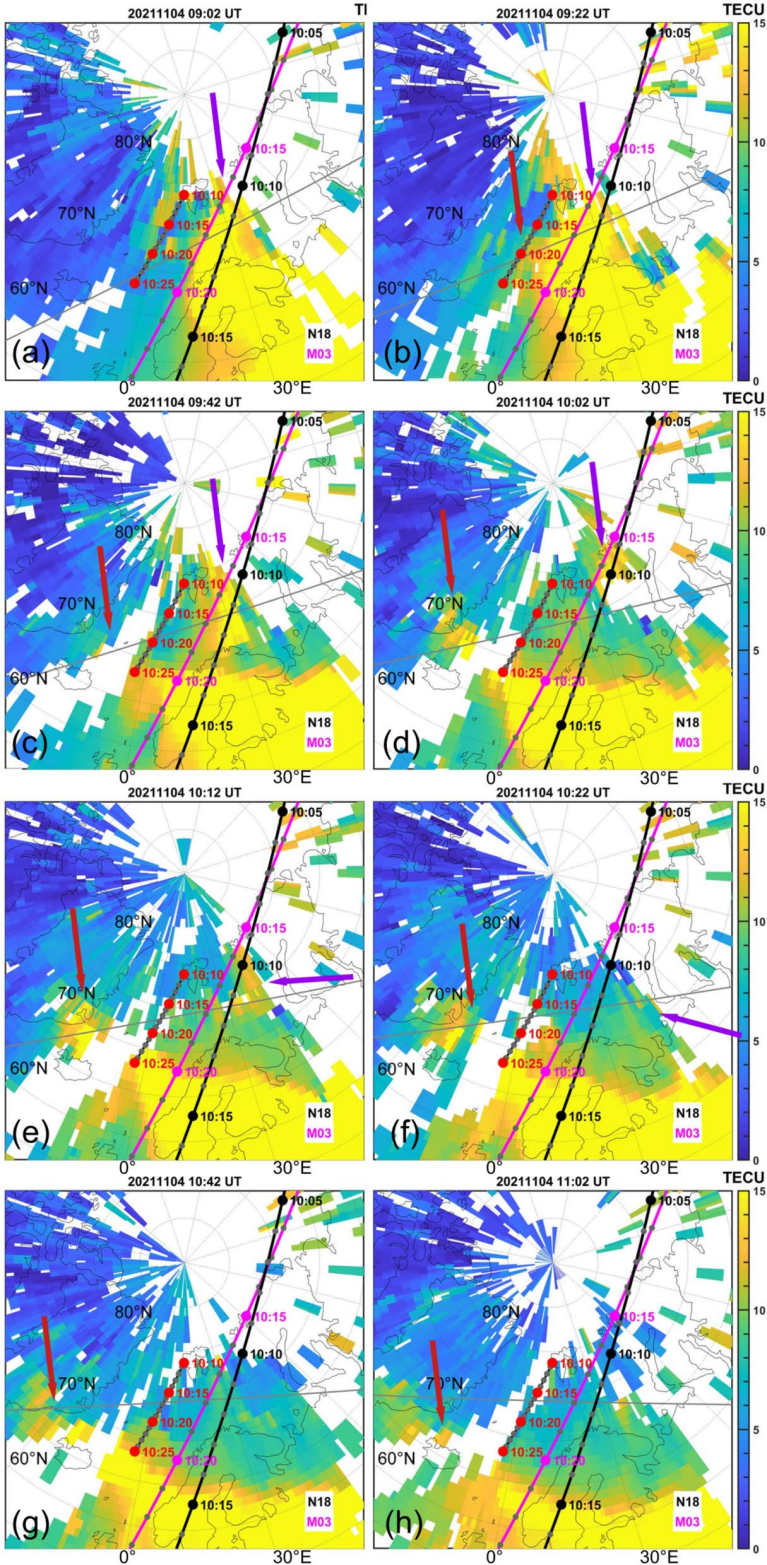
TEC map at different times between 09:02 UT and 11:02 UT obtained from the Madrigal database (Rideout and Coster (2006); Vierinen et al. (2016)), in geographic coordinates. The black lines with grey dots indicate the NOAA-18 trajectory, the magenta lines with grey dots indicate the MetOp-03 trajectory, while the grey lines with red dots indicate the rocket trajectory. The arrows indicate TOI that transverse into the post-noon sector (purple arrows) and the pre-noon sector (red arrows). Note that the images are spaced with 20 min time in between each panel, except for 10:02 UT, 10:12 UT and 10:22 UT. The nearly horizontal grey line indicates the solar terminator

Figure 4 shows TEC maps between 09:02 UT and 11:02 UT obtained through the Madrigal database. The time interval between each panel is 20 min, except for the interval 10:02-10:12-10:22 UT, which is concurrent with the rocket flight time. The black line with grey dots indicates the trajectory of the NOAA-18 satellite, the magenta line with grey dots indicates the trajectory of the MetOp-03 satellite. Precipitating electron data obtained from both satellites can be seen in Fig. 6. In Fig. 4, the grey line with red dots indicates the rocket trajectory. The grey dots along the trajectories indicate the time in between each 5-min interval. The arrows indicate TOI that transverse into the post-noon sector (purple arrows) and the pre-noon sector (red arrows). The fainter grey line that follows nearly horizontally along the graphs is the solar terminator at 350 km for each time, respectively. The plots are presented in geographic coordinates, for completeness and discussion, we additionally present the same plots in magnetic latitude (MLat) and magnetic local time (MLT) coordinates in Appendix A (Fig. 9). In the following description, we give the results in geographic coordinates only to be consistent with the rest of the manuscript.

Panel a of Fig. 4 shows enhanced TEC eastward of approximately $15^{\circ }\hbox {E}$. This enhancement is associated with solar EUV radiation and the corresponding increase in electron density. A TOI is moving poleward in between 15 and $30^{\circ }\hbox {E}$. Note the lack of data points east of $30^{\circ }\hbox {E}$, presented in white.

Panel b shows the development 20 min after panel a. While the TOI that is protruding from the solar EUV enhanced region is growing, an enhancement in the pre-noon sector is developing at around $10^{\circ }\hbox {E}$ between 67 and $73^{\circ }\hbox {N}$.

This enhancement is further traveling towards the dawn-side in panel c at 09:42 UT. Due to the data gaps it remains unclear if the enhancement is moving equatorwards or if denser plasma at lower latitudes is being convected polewards. The duskward protruding TOI detached from the region of enhanced TEC on the dayside in panel c and d (09:42 and 10:02 UT, respectively) and is traveling further over the polar cap towards the night side, as can be observed in panels e-h. Due to the large amount of data gaps in the dusk sector, it becomes difficult to follow the trajectory of the enhancement exactly. The newly formed TOI, indicated by the red arrows, convects further into the pre-noon sector until it reaches up to approximately $70^{\circ }\hbox {N}$ and $30^{\circ }\hbox {W}$ in panel f at 10:22 UT. This is likely due to the shift towards positive $B_y$ values of the IMF as seen in Fig. 2. It becomes apparent that the rocket encounters this newly formed TOI between 10:20 and 10:25 UT. Furthermore, the rocket crosses the terminator at approximately 10:17 UT. Note, that while the pre-noon sector TEC is enhanced due to the convection of the TOI, not all areas equatorward of the terminator may show equal enhancements due to a variety of factors including diurnal variations and data availability in different regions. Due to the high solar zenith angle in November, regions closer to the terminator will also be lower in TEC due to the time it takes for the atmosphere to ionize.

**Fig. 5:**
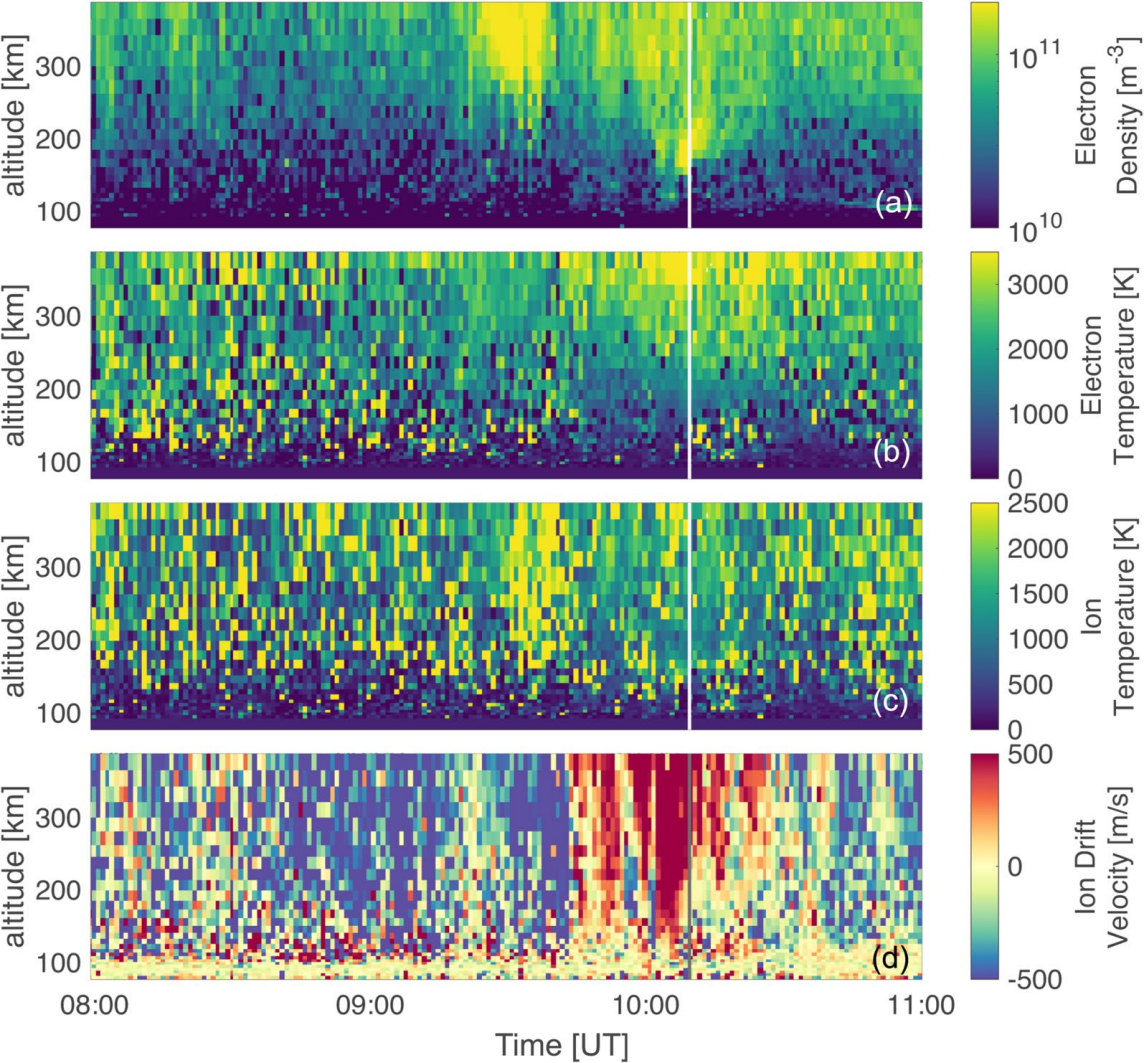
Electron density (**a**), electron and ion temperature (**b** and **c**, respectively) and ion drift velocity (**d**) from the 32 m EISCAT Svalbard Radar (ESR). The figure shows data between 08:00 UT and 11:00 UT. The launch time is indicated by a white vertical line in **a**-**c**, and a grey vertical line in **d**. Note that positive ion drift velocities correspond to a velocity away from the radar

Figure 5 shows the electron density (panel a), electron temperature (panel b), ion temperature (panel c) and ion drift velocity (panel d) between 8:00 UT and 11:00 UT from the 32 m EISCAT Svalbard Radar (ESR), located in Longyearbyen, Svalbard with 60 s resolution. The field of view of the radar aligned close to the rocket trajectory can be seen in Fig. 1. Panel a in Fig. 5 shows a significant increase in electron density around 09:30 UT. As there is no accompanying rise in the electron temperature (see panel b), this structure could be connected to a polar cap patch. Between approximately 09:50 and 10:20 UT patches with enhanced electron density are found at various altitudes. Additionally, panel b in Fig. 5 shows a significant enhancement in the electron temperature after 09:50 UT and for altitudes higher than 350 km. The enhancement in the electron temperature is present until around 10:40 UT, thus all through the rocket flight time. Panel c shows an overall constant ion temperature, except for an enhancement around 09:35 UT, which aligns with the enhancement of the electron density above 200 km altitude. Panel d shows two regions with negative ion drift velocity towards the radar (negative values in blue indicate flow towards the radar, while positive, red values indicate flow away from the radar): the first one between 08:40 and 09:15 UT, the second one between 09:30 and 09:40 UT, which is coinciding with the enhanced ion temperature described prior. A drift velocity towards the radar means a plasma motion with a component in the north-east direction.

The ion drift away from the radar has a maximum speed of 500 m $\hbox {s}^{-1}$, and it is found between 10:00 UT and 10:10 UT down to 150 km altitudes. Until 10:30 UT, the structure is then moving upwards in altitude and is co-located with an enhancement of the electron density with upward motion at around 200 km height between 10:00 UT and 10:10 UT. This structure is occurring simultaneously with the launch of the rocket. Note that the radar field-of-view does not reach the TOI at the needed altitudes to observe said feature in the figure.

**Fig. 6:**
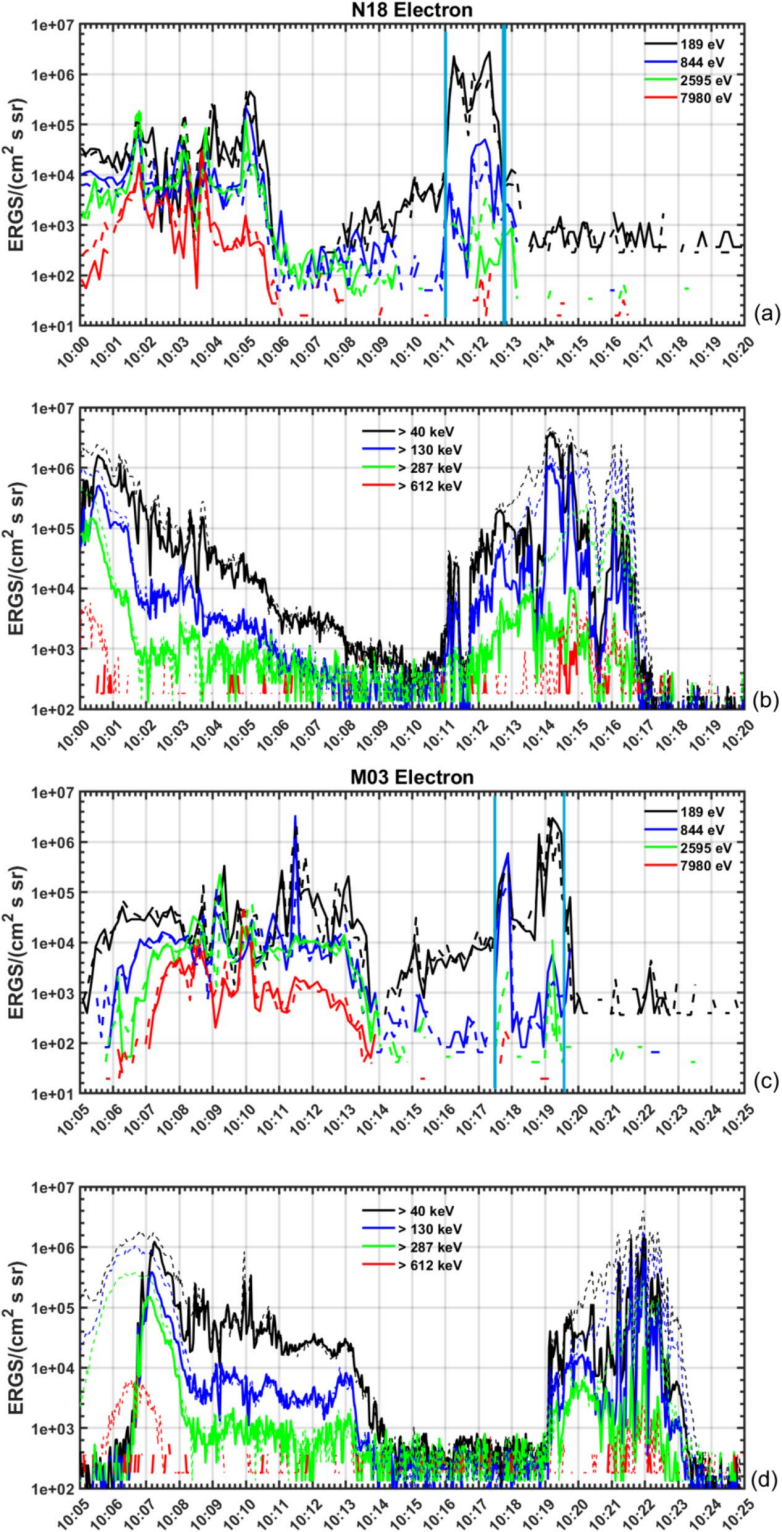
Soft (up to 8 keV) and hard (40–612 keV) precipitating electrons from the NOAA-18 satellite (**a** and **b**, respectively), and from the MetOp-03 satellite (panels **c** and **d**, respectively). The soft electron precipitation (**a** and **c**) are obtained from the $0^{\circ }$ and $30^{\circ }$ TED (solid and dashed lines, respectively). The hard electron precipitation (**b** and **d**) are obtained from the $0^{\circ }$ and $90^{\circ }$ MEPED (solid and dashed line, respectively). The light blue vertical lines indicate two regions of enhanced soft electron precipitation

Figure 6 shows the soft and hard electron precipitation within different energy ranges obtained from the NOAA-18 and MetOp-03 satellites (labeled as N18 and M03, respectively). The energy bands that are swept are centered around the values given in the upper right corner. The soft particle precipitation is obtained by the Total Electron Detector (TED), the solid lines indicate data from the $0^{\circ }$ TED, and thus measuring particles along the magnetic field lines for each energy band, respectively. The dashed lines show data from the $30^{\circ }$ TED. The high-energy electrons are measured by the Medium Energy Proton and Electron Detector (MEPED), the solid lines indicate again data from the $0^{\circ }$ MEPED, the dashed lines indicate data obtained from the $90^{\circ }$ MEPED and thus depict particles that are trapped within the magnetic field configuration. The cyan lines in panel a and c indicate the region where the soft precipitation is increased for visibility reasons.

There is an additional increase in electrons of the <40 keV and >130keV (black and blue lines, respectively), which exhibits similar features as the previously described enhancement of panel a. This enhancement may be an indication of the mapped dayside plasma sheet, but is not subject of this study and may be further investigated in a different work. Furthermore, there is an increase in all bands between 10:00 UT and 10:06 UT. However, this timeframe corresponds to what the satellites are measuring while crossing the duskside auroral region, as seen from the trajectories in Fig. 4. We are mainly interested in events close to the rocket trajectory, which equates to times between 10:10 UT and 10:15 UT. Additionally, we observe an increase in high-energy, or hard particle precipitation (panel b) for the 40–287 keV channels after 10:12 UT. While the field-aligned precipitation ($0^{\circ }$ MEPED, solid lines) decrease again for about 1.5 min around 10:13 UT, the $90^{\circ }$ MEPED data (dashed lines) continue to increase until 10:17 UT. Electrons with lower energies can enter the ionosphere directly into the cusp in between the open and closed field lines, where direct access to magnetosheath plasma exists. Particles bound to the field-lines often are of higher energies, resulting in hard precipitation. Panel c shows an increase in both $0^{\circ }$ and $30^{\circ }$ TED data (solid and dashed lines, respectively) for the 189 and 844 eV channels between 10:17:30 and 10:18:00 UT. This corresponds to similar geographic latitudes that the rocket crossed between 10:17 UT and 10:19 UT (see Fig. 4). The flux drops again between 10:18:00 and approximately 10:18:45 UT. While the 844 eV channel continues to measure a lower flux, the 189 eV channel measures a larger flux between 10:18:45 and 10:19:45 UT. This corresponds to a region between approximately $71^{\circ }\hbox {MLat}$ and $67^{\circ }\hbox {MLat}$, which the rocket crossed between 10:21 UT and 10:23 UT. Panel d shows an increase in hard particle precipitation from both the $0^{\circ }$ and $90^{\circ }$ MEPED between 10:19:10 UT and approximately 10:23 UT.

Note that the longitudinal separation between the rocket and the satellites is at least 500 km, and thus local phenomena obtained by the satellite may not be registered at the rocket’s location and vice versa.

### In situ rocket data: electron density and LEP analysis

**Fig. 7:**
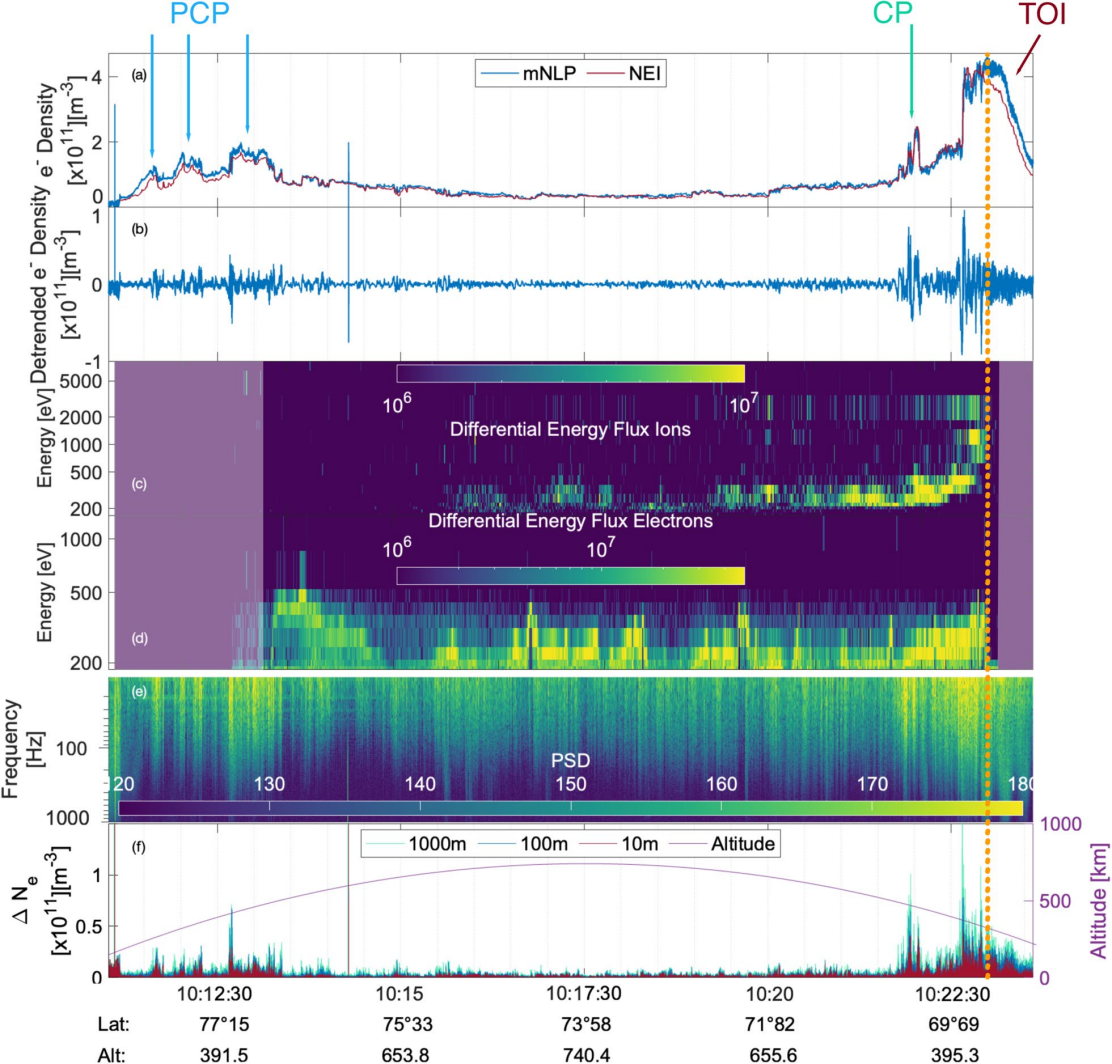
Electron density from the mNLP (**a**, blue line) and NEI (**a**, orange line), the detrended density of the mNLP (**b**), the corresponding spectrogram (**e**), the precipitating ions and electrons from the LEP instruments (**c** and **d**, respectively), and gradients within the detrended electron density for scales of 10, 100 and 1000 m in red, blue and turquoise, respectively (**f**). **f** Also shows the rocket altitude in purple. Panels **a**, **b** and **f** are of the order of $10^{11}$. The arrows indicate the polar cap patches (PCP, blue arrows), the cusp patch (CP, green arrow) and the tongue of ionization (TOI, red arrow). The vertical orange lines indicate the end of the registered particle precipitation

Figure 7 shows the electron density obtained by the mNLP (panel a, blue line) and the electron density obtained with the NEI probe (orange line). Panel b shows the detrended electron density calculated from the mNLP density, detrended with a 3rd order Savitzky–Golay filter using a sliding window of 10 s. The trend was then subtracted from the density as seen in panel a, so that just the smaller scale fluctuations remain in panel b. Panels c and d show the field-aligned downward streaming ions and electrons, respectively, measured by the LEP instruments. The area of collection is in an angle of $30^{\circ }$ around the magnetic field. The greyed out area in the beginning of panels c and d indicates where the voltages of the instruments were raised until the highest voltages were reached; prior to 10:13 UT the instruments were thus not fully functional. The greyed out area at the end of the flight indicates that the instrument had turned off at the end of the flight, thus not collecting data. Panel e shows the spectrogram obtained from the mNLP electron density (blue line, panel a), where the color scale shows the power spectral density that gives a measure of the power present in the signal for a certain frequency. Panel f shows the variations within the detrended electron density for scale sizes between 10 m to 1000 m. The density variations were calculated from the detrended electron density (panel b). For each datapoint (3200 datapoints per second) we subtracted that datapoint from the datapoint that followed 10, 100 or 1000 m later. In order to avoid negative values from the subtraction, we used the absolute value of the result of subtraction. Additionally, panel f shows the altitude of the rocket throughout the flight, indicated in purple.

The rocket encounters three electron density enhancements with strong fluctuations in the first few minutes of the flight between 10:11 UT and 10:13 UT as seen from panel a (indicated as PCP with blue arrows), panels b and e. The density rises from approximately $5\cdot 10^{10}\hbox {m}^{-3}$ in the beginning of the interval up to around $2\cdot 10^{11}\hbox {m}^{-3}$ at the largest density. These enhancements in density are connected to polar cap patches, as the rocket is still located poleward of the cusp and the enhancements are approximately twice the background density. Two further density increases are found towards the end of the flight, the first one just before 10:22 UT (indicated with CP in green in panel a), the second, larger one starting at 10:22:30 UT and lasting throughout 10:23:30 UT (indicated as TOI in purple in panel a). The enhancement around 10:22 UT is a brief increase of density from roughly $1\cdot 10^{11}\hbox {m}^{-3}$ to $2.4\cdot 10^{11}\hbox {m}^{-3}$, while the second, larger enhancement rises from $1.8\cdot 10^{11}\hbox {m}^{-3}$ to $4.7\cdot 10^{11}\hbox {m}^{-3}$. While the first of these two enhancements can be connected to a cusp patch (CP), an enhancement with similar characteristics to a PCP but located in the cusp, the second, larger enhancement corresponds to the region where the rocket enters the poleward side of the TOI, as seen in Fig. 4. Similar to the enhancements in density at the beginning of the flight, both increases in density show strong irregularities, as seen in panels b and e and f. During the remaining part of the flight only small density variations are seen.

Panel c shows the downward precipitating ions, following along the magnetic field lines. No ions are detected until approximately 10:15:50 UT, when particles with less than 500 eV were measured. The flux increases briefly at 10:20:10 UT and then again about a minute later. At 10:22:50 UT, a sharp increase in ion energy up to 3 keV can be observed. After that time no more ions are detected. This rapid increase in ion energy can be connected to ion dispersion resulting from observation within low latitudes, which has been observed with sounding rockets before (Tanaka et al. 2003; Reiff et al. 1977). From the increase in soft particle precipitation observed from the NOAA-18 and MEPED satellites shown in Fig. 6, and the onset of precipitating ion data, we conclude the poleward boundary of the cusp to be at approximately $74^{\circ }\hbox {N}$, which the rocket crosses at about 10:16:15 UT. Panel d shows, similar to panel c, the downward precipitating electrons. Again, the beginning of the flight shows no detected precipitating electrons, until 10:12:30 UT. Thus electron precipitation is detected 2-3 min earlier in the flight than precipitating ions. Note that the instrument voltage was still raised until 10:13:05 UT. The precipitating electrons have energies up to 600 eV, and several inverted-V structures can be observed at about 10:16:45 UT, 10:19:40 UT and 10:21:40 UT with peak energies of a few hundred eV. After 10:21:40 UT the electron flux and corresponding energy increase, before they suddenly fall down to an energy of 200 eV at around 10:22:50 UT, where no further particles are detected around 10:23:15 UT. Note, that the LEP instrument stopped collecting data around the same time. The ion precipitation already stopped at this point, it is, however, not possible to make further assumptions about the end of the electron precipitation. Furthermore, it is worth mentioning that the electron densities shown in panel a are solely derived from the mNLP and NEI instruments, respectively, and are thus not reliant on the LEP data.

**Fig. 8:**
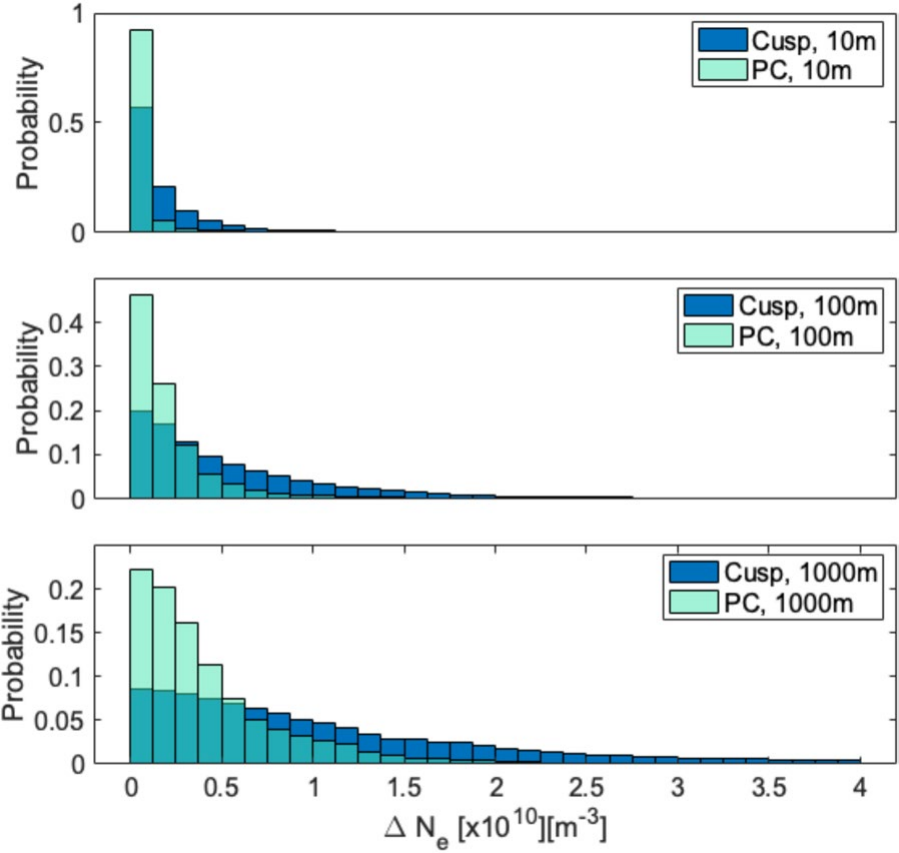
Histogram of the variations in electron density (Figure 7, panel f). The events in the polar cap (PC) include the three PCP and analyses the variations between 10:11:05 UT and 10:12:25 UT. The event in the cusp includes the CP and TOI and analyses data in between 12:21:40 UT and 10:23:30 UT. The histogram shows the probability on the y-axis and the strength of the fluctuations on the x-axis for the three scale sizes (10, 100 and 1000 m)

**Fig. 9:**
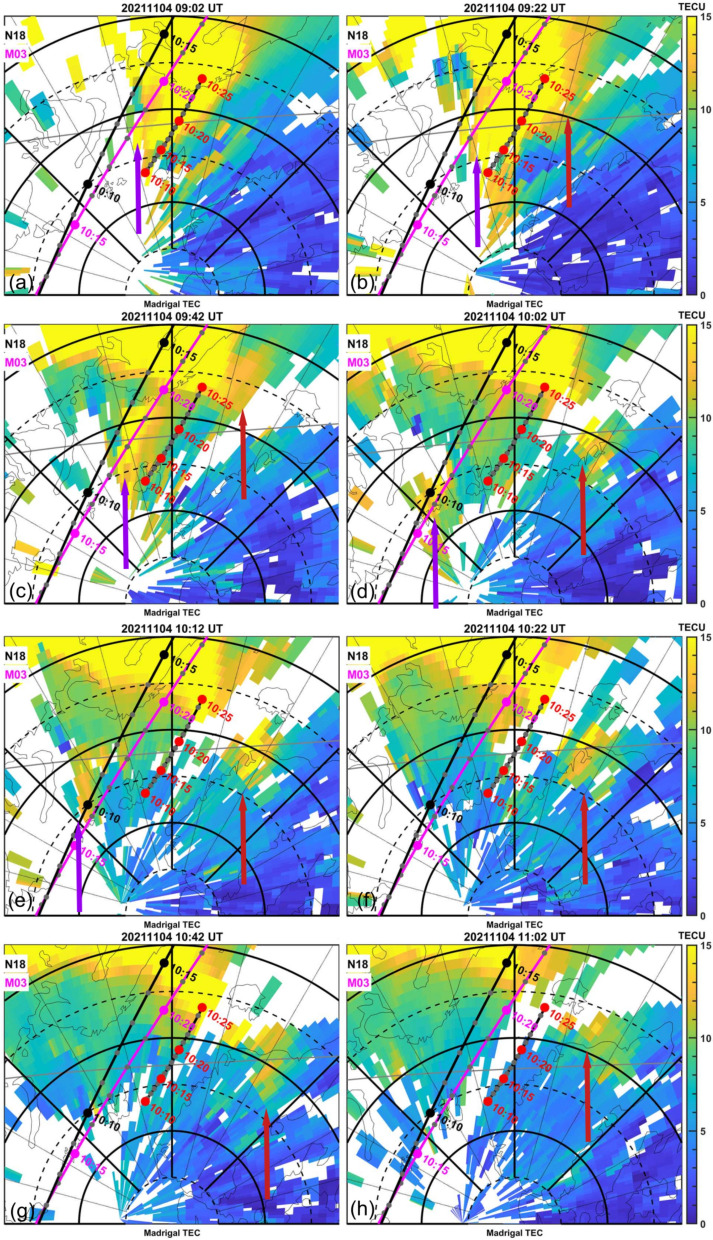
TEC map at different times between 09:02 UT and 11:02 UT obtained from the Madrigal database, in magnetic coordinates. The black line with grey dots indicates the NOAA-18 trajectory, the magenta line with grey dots indicated the MetOp-03 trajectory, while the grey line with red dots indicates the rocket trajectory. The arrows indicate TOI that transverse into the post-noon sector (purple arrows) and the pre-noon sector (red arrows). Note that the images are spaced with 20 min time in between each panel, except for 10:02 UT, 10:12 UT and 10:22 UT. The nearly horizontal grey line indicates the terminator. The graphs are given in magnetic latitude/magnetic local time

Panel f shows the gradients within the electron density obtained from the detrended density of the mNLP. Between 10:11:30 UT and 10:13:30 UT enhancements within all scales are observed, especially on the edges of the polar cap patches, as seen in panel b. While enhancements in all scales occur, the 100 and 1000 m scales are slightly higher than the 10 m scales, as it is expected. Towards the end of the flight, between 10:21:40 and 10:23:40 we also observe enhancements within all scales. While the 10 m scales show slightly higher values in this region than in all other regions throughout the flight, the 100 and especially the 1000 m scales exhibit much higher values, especially co-located with the cusp patch at 10:21:30 UT and the poleward edge of the TOI at 10:22:40 UT. Note that, while both the PCP at the beginning of the flight and the CP and TOI towards the end of the flight occur at comparable altitudes, as well as similar absolute densities for the PCP and CP, the overall enhancement of density variations within the scales is considerably larger for the CP and TOI gradients. In order to quantify the variations within the electron density, we have included a histogram over the strength of fluctuations comparing structures in the polar cap and cusp in Fig. 8. In order to construct the histogram, we focused solely on the enhancements in electron density that the rocket encountered in the polar cap and cusp. The PCP were encountered by the rocket between 10:11 UT and 10:13 UT and form the interval used for the analysis of the polar cap histogram. The CP was encountered just before 10:22 UT, while the TOI was encountered between 10:22:30 UT to just after 10:23:15 UT when the density decreases. Thus, the analysis of the cusp histogram was conducted for the time frame 10:21:45 to 10:23:15 UT.

The histogram shows that there are stronger fluctuations in the electron density on all scale sizes within the cusp TOI and CP in comparison to the PCP, but that small-amplitude fluctuations are twice as common in the polar cap for 100 m and 1000 m scales. Additionally, it is interesting to see that the probability for small-scale structures (10 m) in the PC is nearly doubled in comparison to the cusp. Note, that the regions compared in the histogram do not cover the whole area where the rocket was located in either the PC or the cusp. The histogram rather compares the structures found in the two regions (namely PCP for the PC and CP and TOI for the cusp). The times given in the figure caption do thus not align with the time the rocket enters and exits the cusp.

Noteworthy is also the comparison between the mNLP and NEI probes (panel a). During the intervals where little density fluctuations are observed, the two probe signals are very closely related to each other. This changes for regions with higher density fluctuations, especially within the PCP and the TOI. While the factor between the densities measured is generally low (below 1.5), it becomes apparent that specific alterations within the electron density seem to affect the accuracy of the probes.

## Discussion

The main emphasis of this study is to provide the background conditions for the launch of the SS-520-3 sounding rocket, giving context for the launch and future studies. Additionally, we provide an investigation of the electron density structures and electron and ion particle precipitation obtained from the rocket. In order to help the flow of reading, the main events encountered by the rocket and obtained from other instruments used in this study are listed in Table 4 with corresponding timestamps.

**Table 4:** Events obtained from the rocket and other instruments used in this study

Time [UT]	Event	Source
08:40 - 09:15	Ion flow with north-east component	EISCAT
09:10-09:20	IMF $B_x$, $B_y$ and $B_z$ change	OMNI
09:30-09:40	Ion flow in north-east direction	EISCAT
09:30	Increase in electron density above 200 km altitude	EISCAT
09:40 - 10:30	Ion flow with south-east component	EISCAT
09:50-10:20	Sporadic electron density enhancements above 150 km altitude	EISCAT
10:09	IMF $B_x$ and $B_z$ change	OMNI
**10:09:25**	**Launch**	
10:11-10:13	Polar cap patches	mNLP
10:12:30	Electron precipitation onset	LEP
10:15:50	Ion precipitation onset	LEP
10:16:15	Rocket crosses poleward boundary of the cusp	Various
10:16:45	inv v	LEP
10:17:00	Rocket encounters strong scintillations	GNSS receivers
10:19:40	inv v	LEP
10:21:40	inv v	LEP
10:21:40	Rise in electron energy	LEP
10:22:00	Cusp patch	mNLP
10:22:30	TOI	mNLP
10:22:40	Rise in ion energy	LEP
10:23:00	Drop in electron energy	LEP
10:23:00	End of ion precipitation	LEP
10:23:15	End of electron precipitation	LEP
10:23:15	Rocket crosses equatorward boundary of the cusp	Various

The bold entries in row 8 indicate the launch time

The rocket was launched into the main phase of an ongoing geomagnetic storm (Regi et al. 2022), with Sym-H indices of below -100 nT, as seen in Fig. 2. The rocket encountered high phase scintillations around 10:17 UT, just equatorward of $75^{\circ }\hbox {N}$, as seen in Fig. 3. Simultaneously, the TEC values obtained from the scintillation receivers as seen in Fig. 3 are also enhanced. This coincides with the Madrigal TEC values as seen in Fig. 4.

As seen in Fig. 6, the NOAA-18 satellite encounters an enhancement of soft particle precipitation between 10:11 UT and 10:13 UT (as seen in panel a). Additionally, the MetOp-03 satellite measures soft particle precipitation between 10:17:30 UT and 10:19:30 UT (panel c). Particles with such low energy (below 1 keV) have their origin within the magnetosheath and can directly enter the ionosphere in the cusp region. Hard particle precipitation, or particles with higher energies, can gyrate around the closed field lines until they have sufficient energy to leave the confinement through the loss-cone. These trapped particles are measured by the NOAA-18 satellite from approximately 10:12:30 UT on and by the MetOP-03 satellite from approximately 10:19:10 UT on. The observations show that the open-closed field line boundary, and thus the equatorward side of the cusp, is located between around approximately 10:12 and 10:13 UT, as measured by the NOAA-18 satellite, and to approximately 10:19:30 UT for the MetOp-03 satellite. This sets the equatorward boundary to around $69.5^{\circ }\hbox {N}$. The rocket encounters these latitudes at approximately 10:23:15 UT. While this is generally consistent with the observations from the rocket in situ measurements, it is worth mentioning that the LEP instrument stopped taking reliable data after 10:23:15 UT as well. Moreover, discrepancies between observations from the rocket and satellites are to be expected. Indeed, the ionosphere is highly dynamic. Furthermore, about 500 km in longitude separate the spacecrafts at their closest point. From the particle precipitation data we thus assume that the rocket entered the cusp region around 10:16:15 UT or at approximately $74^{\circ }\hbox {N}$. Magnetosheath particles precipitating down into the cusp usually consists of low energy ions (50 eV to 5 keV) and low energy electrons around 50 eV (Newell and Meng 1988). Both precipitating ions and electrons are detected. Additionally, the precipitating electrons show several inverted V-structures with energies of up to a few hundred eV. These low-energy structures within electron precipitation are often found within the cusp and further confirm that the rocket encountered the cusp (Newell et al. 1991; Tanaka et al. 2003; Moen et al. 2012). Note also, that the inverted v-structures encountered between approximately 10:16 and 10:21 UT do not seem to correspond to enhancements in the electron density. As the rocket is flying at high altitudes above 500 km during these times, it is likely that the particles’ energy is being recorded, while the particles do not lead to a significant change in density, as they travel to lower altitudes. Additionally, the electron density as seen in panel 7a has been plotted semi-logarithmically for comparison to study the smaller fluctuations in the central cusp which are naturally of lower amplitude as the density will diminish at higher altitudes. During this comparison, no significant or distinct correlating features with enhancements of the particle precipitation of the LEP have been found.

From the lower energy in precipitating electrons at 10:23 UT and the absence of ions we conclude that the rocket passed a newly reconnected, open field line. As discussed above, the rocket entered the cusp at 10:16:15 UT, and we set the equatorward boundary to approximately 10:23:15 UT, as measured from NOAA-18 data at 10:13 UT and the MetOP-03 data at 10:19:30 UT. With the newly reconnected field line the rocket passes towards the end of the flight, the equatorward boundary shifts further towards the south. Note that this spans the thickness of the cusp to approximately $6^{\circ }$ in geographic latitude, which is larger than what has been observed before (Newell and Meng 1988). Note again, that the LEP instrument is not collecting data after 10:23:15 UT and thus no further conclusions about precipitating particles can be made after this point. It is, however, noteworthy that the stopping of registered ion precipitation and the loss of electron energy occurred before the shutoff of the LEP instrument, and may thus still give an indication about the cusp boundary.

Simultaneously, a strong increase in ion energy within the precipitating particles is observed, as seen in Fig. 7, panel c from 10:22:00 UT onward. A similar phenomenon has been observed earlier from the SS-520-2 rocket, and it has been attributed to the invariant latitude variation of the rocket trajectory. The rocket encounters more highly energetic ions at lower latitudes caused by an anti-solar convection pattern due to dayside reconnection (Tanaka et al. 2003; Reiff et al. 1977).

Before 09:00 UT the IMF $B_y$ component was weakly negative alongside a $B_z$ with values of around -10 nT. This leads to a configuration where the convection cell on the post-noon expands into the pre-noon sector and pushes the cusp accordingly into the post-noon sector as well (Jin 2016). A TOI formed within the post-noon sector close to magnetic noon and stretched into the nightside until the IMF changed around 09:00 UT, with a strongly positive $B_y$ and a neutral $B_z$, as seen in Figure 2 d. The strong positive $B_y$ changed the configuration of the convection cells, moving the cusp further into the pre-noon sector. Due to the change in convection cell configuration high-density dayside plasma can be effectively transported, and the resulting TOI is convecting over the pre-noon sector into the nightside. The convection of the TOI into the pre-noon sector can be seen well in Fig. 4, panels c-h. These results are also consistent with the regions of increased ion drift velocity that were observed from the EISCAT radar during launch time, seen in Fig. 5 d. While the line-of-sight of the EISCAT radar does not directly reach the altitude and location of the TOI, it is possible to assume that the ionospheric convection that is observable is similar to that of the TOI. As seen from Fig. 1, the radar was aligned south-west intersecting the rocket trajectory. The TOI that was convecting towards the nightside over the duskside before 09:40 UT (see again Fig. 4 a and b) has a velocity component in the north-east direction which corresponds to a flow towards the radar. Two regions of such kind of flow were observed by the radar between 08:40 and 09:40 UT. Additionally, the radar registers a plasma convection in the south-west direction between 09:50 and 10:30 UT, seen in Fig. 5 a. The TOI that forms after 09:20 UT (indicated by the red arrow in Fig. 7 b onward) due to the change in IMF travels towards the dawn-side and thus towards the west, indicating a velocity component into the south-west direction.

At the beginning and towards the end of the flight the rocket encountered several enhancements in the electron density. The first enhancements intersected by the rocket were located poleward of $75^{\circ }\hbox {N}$ and can thus be connected to PCP as they were located poleward of the cusp. The density rose approximately by a factor of two. The fluctuations within this region were generally high, and density gradients showed an enhancement in all analyzed scales, especially on the edges of the PCP.

Towards the end of the flight time at 10:22 UT and at $70^{\circ }\hbox {N}$, the rocket encountered another electron density enhancement. As this enhancement was located within the cusp, the rocket encountered a cusp patch (CP). The patch was characterized by large density variations at all three scales considered, though kilometer scales were the strongest. Additionally, the patch showed the largest enhancement within meter-size scales.

The last enhancement in electron density is co-located with entering the newly formed TOI. The TOI is associated with an enhancement in density variations throughout all scales, though the kilometer-sized scales are especially raised within this region. The strongest enhancements throughout the flight within all scales are found just on the poleward edge of the TOI and could be favored by the KHI arising from the flow shear between the convecting TOI and the background plasma. The plasma within the TOI is strongly structured up until 10:23:00 UT and then is smoothed out. The structured region of the TOI starting at 10:22:30 UT is co-located with particle precipitation of both electrons and ions. Once the rocket crosses the equatorward boundary of the cusp, indicated by the orange line in Figure 7, the measured electron density becomes smoother. The largest enhancements in the kilometer-sized scales in comparison to scales on meter-sizes are observed within the TOI. Overall, while both the PCP and the CP and TOI are encountered at similar altitudes, the structuring and irregularities within the electron density are significantly larger inside the structures within the cusp, e.g., the CP and the part of the TOI located within the cusp. Note that the terminator was crossed by the rocket at approximately 10:17 UT, in a region of little density fluctuations and several minutes before the largest density fluctuations are observed. Crossing the terminator has thus, as expected, little influence on these measurements, as crossing the terminator does not yield abrupt changes in the plasma.

Overall, the electron density fluctuations show very little variations in between the regions where the rocket encounters PCP and the CP, so between 10:13:30 and 10:21:30 UT. During this entire interval the rocket encounters precipitating electrons. In the second half of the interval, precipitating ions are also measured. This is likely related to the rocket altitude, which for this region is between 500 and 742 km. From the scintillation data presented in this study, we know that the rocket entered a region with high phase scintillation at 250 km altitude after approximately 10:17 UT. Phase scintillation has been shown to be caused by structures within the electron density of several hundred meters up to a few kilometers (Kintner et al. 2007; Jin et al. 2017). Fluctuations on these scale sizes would be visible in the variations in the electron density within the corresponding scale sizes. We thus conclude that strong fluctuations within the electron density, if existing throughout the flight, may not be registered by the rocket, as the irregularities are localized within the E or lower F-region at altitudes several hundred kilometers lower than that of the rocket.

## Conclusion

The SS-520-3 sounding rocket was successfully launched into the cusp ionosphere. We utilized electron density data from both the multi-needle Langmuir probe (mNLP) and an impedance probe (NEI). The overall difference between densities observed with both probes is very low within regions of little fluctuations in the electron density. When the rocket encounters higher density fluctuations, especially within the PCP and TOI, the discrepancy widens. While overall low (up to a factor of 1.5), different structures within the plasma seem to affect the accuracy of the mNLP, further studies on this are needed to get a better understanding.

The launch of the sounding rocket fell into the main phase of a geomagnetic storm. EISCAT data showed two regions of enhanced plasma motion towards the radar (with a north-east drifting component) before 09:40 UT. Additionally, a drift velocity of up to 500 m $\hbox {s}^{-1}$ away from the line of sight reaching up from 100 km altitude during launch time was observed between 09:50 and 10:30 UT. Moreover, an upward flowing electron density enhancement at 150 km and electron temperatures up to 4000 K at 350 km altitude were observed at the time of launch. Data from scintillation receivers around Svalbard showed that the rocket encountered an enhancement in phase scintillation index above 0.4 equatorward of $75^{\circ }\hbox {N}$. At the same time VTEC values were enhanced. This was further confirmed by Madrigal TEC data and the analysis of soft and hard electron precipitation obtained from the NOAA-18 satellite. The $0^{\circ }$ and $30^{\circ }$ TED analyser showed that the satellite encountered soft particle precipitation between 10:11 UT and 10:13 UT and hard particle precipitation from 10:12:30 UT onward. We thus concluded the equatorward boundary of the cusp to be about roughly $68^{\circ }\hbox {N}$ during the pass of the satellite. When the rocket flew through the cusp, however, we observe a sudden drop in electron precipitation energy accompanied by a lack of ion precipitation that we connect to a newly opened field line at 10:23 UT where the electron precipitation energy (Figure 7, panel d) drops. From the enhancements in phase scintillation, TEC and the measurement of precipitating electrons and ions we thus conclude that the rocket entered the cusp around 10:16:15 UT at $74^{\circ }\hbox {N}$ and that the rocket exited the cusp towards the end of the flight at $69.5^{\circ }\hbox {N}$. This is an example of a cusp that is located more equatorward and spanning over a larger range of latitudes than usual. The more equatorward position is to be expected for enhanced conditions during the main phase of a geomagnetic storm, which may also be linked to the expansion of the cusp over a larger range of latitudes. Ionospheric dynamics within the cusp have been shown to cause severe detrimental influence on GNSS signals. With events like these it becomes more important to investigate the dynamics within the cusp and their impact on trans-ionospheric radio signals.

The rocket encountered polar cap patches in the first part of the flight poleward of $75^{\circ }\hbox {N}$. The PCP were associated with a rise within gradients on all scale sizes and intense electron density fluctuations, especially on the edges of the PCP. The kilometer-size scales were enhanced the strongest, as anticipated from PCP; however, the difference between enhancement in meter and kilometer sizes is small. Towards the end of the flight the rocket encountered both a cusp patch and entered a newly formed TOI convecting into the pre-noon sector. The patch and TOI were associated with a strong rise in gradients within all scales, similar to the PCP. However, while both events were encountered at similar altitudes as the PCP, the enhancements in both the density fluctuations and gradients were significantly higher than for the PCP. The strongest overall rise within the gradients was observed just on the poleward edge of the TOI with a strong increase in all scales. This could be connected to the convection of the TOI with respect to the background plasma, leading to instabilities arising from the flow shear, like the KHI. Additionally, the strongest enhancements within kilometer sizes in comparison to meter scales was found within the TOI.

Little density fluctuations were observed between $77^{\circ }\hbox {N}$ and $71^{\circ }\hbox {N}$. The rocket encounters regions associated with scintillations mapped onto 250 km altitude. As scintillations are connected to small-scale fluctuations within the electron density, these should be visible within the calculated electron density gradients. We thus conclude that small-scale structures are located at lower altitudes than that of the rocket for a part of the flight and are thus not measured.

## Data Availability

The datasets used and/or analyzed during the current study are available from the corresponding author on reasonable request.

## Appendix

See Fig. 9.

## Abbreviations

OCB: Open-close fieldline boundary
PCP: Polar Cap Patch
CP: Cusp Patch
TOI: Tongue-of-Ionization
KHI: Kelvin–Helmholtz Instability
GDI: Gradient-Drift Instability
IMF: Interplanetary Magnetic Field
EUV: Extreme Ultraviolet
GNSS: Global Navigation Satellite Systems
MLat: Magnetic Latitude
MLT: Magnetic Local Time
mNLP: multi-needle Langmuir Probe
NEI: Number density measurement of Electron by Impedance
LEP: Low Energy Particle
FFT: Fast Fourier Transform
ESA: Electrostatic Analyzer
MCP: micro-channel plate
EISCAT: European Incoherent Scatter
TEC: Total Electron Content
VTEC: Vertical TEC
TED: Total Electron Detector
MEPED: Medium Energy Proton and Electron Detector
GSM: Geocentric Solar Magnetospheric
